# Within-sample variation in snowshoe hare faecal glucocorticoid metabolite measurements

**DOI:** 10.1093/conphys/cox068

**Published:** 2017-12-08

**Authors:** Diana J R Lafferty, Alexander V Kumar, Sarah Whitcher, Klaus Hackländer, L Scott Mills

**Affiliations:** Fisheries, Wildlife, and Conservation Biology Program, Department of Forestry and Environmental Resources, North Carolina State University, Raleigh, NC 27695, USA; Wildlife Biology Program, University of Montana, Missoula, MT 59812, USA; Department of Biology, Northern Michigan University, Marquette, MI 49855, USA; Institute of Wildlife Biology and Game Management, University of Natural Resources and Life Sciences, Vienna (BOKU), Gregor-Mendel-Str. 33, 1180 Vienna, Austria; Office of Research and Creative Scholarship, University of Montana, Missoula, MT 59812, USA

**Keywords:** cortisol, glucocorticoids, lagomorph, non-invasive, power analysis, stress physiology

## Abstract

Faecal glucocorticoid metabolite (FGM) concentrations are used increasingly as a non-invasive measure to index physiological stress experienced by diverse taxa. However, FGM may not be evenly distributed throughout a faecal mass or faecal pellet group. Moreover, within-sample variation in FGM measurements associated with different sampling and/or processing techniques is rarely reported despite potentially having important implications for inferring stress levels in free-ranging wildlife. Using a captive collection of snowshoe hares (*Lepus americanus*), we (i) assessed repeatability of FGM measurements (i.e. precision) from two processing techniques (measurements derived from dividing whole pellet groups into equal proportions prior to processing [G1], measurements from subsamples derived from thoroughly homogenized whole pellet groups [G2]) and (ii) conducted a power analysis to estimate sample-size requirements for detecting statistically significant differences in FGM concentrations at a population level. Our results indicate that the mean percent coefficient of variation (%CV) for within-sample FGM variation was slightly higher for G1 (%CV = 35, range 13.45–65.37) than for G2 (%CV = 23, range 7.26–47.94), though not statistically significant (two sample *t*-test, *n* = 8, *t* = 1.57, *P* = 0.16). Thus, FGM is relatively evenly distributed within snowshoe hare faecal pellet groups. However, subsampling from homogenized whole pellet groups may be more appropriate when the sampling time frame is less controlled (e.g. multiple defecation events) because a subsample derived from a homogenized whole pellet group might be more representative of the animal's ‘average’ physiological state compared to FGM concentrations derived from a few haphazardly selected faecal pellets. Power analysis results demonstrated the importance of *a priori* consideration of sample sizes. Relatively small effect sizes (e.g. <20%) may require sampling that is logistically and/or cost prohibitive. Yet for many situations of ecological or conservation interest, treatment effects may be substantial (>25%) and thus moderate sample sizes may be sufficient for testing research hypotheses regarding changes FGM concentrations.

## Teaser

Measures of stress hormone levels, specifically glucocorticoids and their metabolites, are increasingly used to index the stress burden experienced by wildlife. However, within-sample variation may affect interpretations of results. Here we quantified within-sample variation in faecal cortisol metabolite concentrations and conducted a power analysis to demonstrate sample-size requirements for detecting meaningful differences in stress hormone levels in wildlife populations following an environmental perturbation.

## Introduction

A major focus of conservation physiology is to identify the causes and consequences of adrenocortical activity (hereafter stress) in wildlife populations and to determine whether the stress responses of individuals scales up to effect population-level health and dynamics. As such, measures of glucocorticoid (GC) hormones (i.e. cortisol or corticosterone depending upon the species) or their metabolites are being used increasingly to index stress in free-ranging and captive wildlife and as a biomarker to infer population-level health (Möstl and Palme, 2002; Millspaugh and Washburn, 2004; Palme *et al.*, 2005; Sheriff *et al.*, 2011a; Goymann, 2012; Dantzer *et al.*, 2014). For example, measures of GCs or their metabolites can provide quantitative information linking individual stress responses to environmental perturbations associated with anthropogenic disturbances (Tarlow and Blumstein, 2007; Creel *et al.*, 2013a), climate change (Romero and Wikelski, 2001, 2010), spatial-temporal variability in food availability (Kitaysky *et al.*, 1999, 2007), the social competitive environment (Bryan *et al.*, 2013, 2014; Creel *et al.*, 2013b) and predation risk (Monclús *et al.*, 2009; Sheriff *et al.*, 2009b). Further, GC concentrations and their metabolites have been linked to variation in reproductive output and differences in population performance in diverse taxa (Creel *et al.*, 1997; Barrett *et al.*, 2002; Kitaysky *et al.*, 2007; Charbonnel *et al.*, 2008; Sheriff *et al.*, 2009b, 2010b; Scarlata *et al.*, 2012). Thus, measures of GCs have tremendous potential to enhance sustainable wildlife management strategies (e.g. conservation initiatives) and to improve health monitoring of both captive and free-ranging populations (Möstl and Palme, 2002; Millspaugh and Washburn, 2004; Palme *et al.*, 2005; Sheriff *et al.*, 2011a; Goymann, 2012; Dantzer *et al.*, 2014).

Although measures of GCs and their metabolites can be obtained from diverse sources (e.g. blood, urine, saliva, hair), faeces may be the most reliable matrix for indexing stress via measures of faecal glucocorticoid metabolite (FGM) concentrations for wild populations (Millspaugh and Washburn, 2004; Palme, 2005; Sheriff *et al.*, 2010a, 2011a; Dantzer *et al.*, 2014). For example, faeces collection is efficient and cost-effective relative to other non-invasive stress hormone sampling techniques (i.e. hair cortisol extraction) (Möstl and Palme, 2002; Millspaugh and Washburn, 2004; Palme *et al.*, 2005; Sheriff *et al.*, 2011a; Goymann, 2012; Dantzer *et al.*, 2014). Sampling faeces can eliminate the potential confounding effects of hormonal habituation due to repeated capture and handling of the same individuals when assessing long-term stress within a population (Cyr and Romero, 2009). Further, FGM concentrations reflect an integrated measure of circulating GC levels, thereby providing an excellent matrix from which long-term stress can be indexed (Harper and Austad, 2000; Sheriff *et al.*, 2010a; Palme, 2012). And while a variety of factors (e.g. abiotic, biotic) affect measured FGM concentrations, these concerns are outlined in the literature and can often be mitigated with appropriate study design and standardized faecal sample processing techniques (Millspaugh and Washburn, 2004; Goymann, 2005, 2012; Touma and Palme, 2005; Sheriff *et al.*, 2011a). However, two important aspect of study design regarding the use of non-invasive sampling of faeces that have received little attention are the repeatability of FGM measurements from a single sample (i.e. precision) and sample-size requirements needed for being able to detect meaningful differences (e.g. 10%, 20% and 40%) in FGM concentrations at a population level.

Snowshoe hares (*Lepus americanus*) are an excellent study species for examining within-sample variation in FGM concentration. First, the use of 11-oxoetiocholanolone enzyme immunoassay (EIA) has been rigorously tested (i.e. dexamethasone suppression test, adrenocorticotropic stimulation test) and found to reliably detect temporal changes in snowshoe hare adrenocortical activity (Sheriff *et al.*, 2009a, 2009b, 2010a, 2011b). Moreover, snowshoe hare plasma GC levels and FGM concentrations are concordant over time (Sheriff *et al.*, 2010a), indicating that snowshoe hare faecal pellets are a suitable non-invasive biological matrix for indexing an individual's stress burden. Research also shows that fresh snowshoe hare pellets represent the physiological state of the individual 8–12 h prior to defecation (Sheriff *et al.*, 2009a), thereby allowing for a sampling frame that can account for the confounding effects of variation in FGM concentrations due to diel hormonal fluctuations associated with the circadian rhythm (Rich and Romero, 2001; Carere *et al.*, 2003; Sheriff *et al.*, 2009a). Further, snowshoe hares typically defecate while foraging, producing variable amounts of faeces in the form of pellets that are easy to subsample because the pellets are not accumulating in a latrine. Thus, using a captive collection of snowshoe hares our objectives were to (i) assess the repeatability of FGM measurements (i.e. precision) from two processing techniques (FGM measurements derived from dividing whole pellet groups into equal proportions prior to processing [G1]; FGM measurements from subsamples derived from thoroughly homogenized whole pellet groups [G2]) and (ii) conduct a power analysis to estimate sample-size requirements to detect differences (e.g. 10%, 20% and 40%) in FGM concentrations at a population level following a perturbation.

## Materials and methods

### Animals and housing

Eight wild-caught adult snowshoe hares were used for this experiment. Animals were live-captured in the Mt. Baker-Snoqualmie National Forest in Washington, USA (four females, four males) using Tomahawk traps (Tomahawk Live Trap Co., Hazelhurst, WI, USA). Animals were transported to North Carolina State University College of Veterinary Medicine (NCSU CVM) and housed in environmental rooms that replicate weekly 30-year average temperature and photoperiod from their capture location. Daytime and nighttime temperatures in the environmental rooms ranged between −1°C and −4°C (winter) during the sampling period. Animals were housed individually in wire mesh cages (121.9 cm width × 60.9 cm depth × 73.6 cm height) with a wire mesh floor. The cages included an acrylic hide box (30.5 cm width × 60.9 cm depth × 73.6 cm height) that served as a visual barrier between animals, as well as an edible grass hut to hide in or sit atop. Premium natural adult rabbit food (Sherwood Pet Health, Logan, UT, USA) and water were available ad libitum. Capture, handling, transport, husbandry and sample collection procedures were approved by NCSU Institutional Animal Care and Use Committee (Protocol 14-069-0).

### Faecal sample collection

Faecal pellets fell through the wire mesh floor and were collected in a tray underneath each cage. Faecal samples were collected over a 2-day period (24–25 December 2015), resulting in two samples per individual with each sample consisting of the total pellets accumulated (faecal pellet group) over a single 10-h sampling frame. To acquire these samples, the collection tray beneath each animal's cage was cleaned between 2200 h and 2220 h, lined with absorbent paper, and the following morning all faecal pellets were collected for each animal between 0800 h and 0820 h; care was taken to avoid pellets exposed to urine. During collection, we placed pellet samples in pre-labelled plastic bags and stored the samples at −20°C within 10 min of collection. This 10-h sampling frame was selected to mimic a plausible period over which animals may be held in traps during field-based operations, and from which faecal pellets may be collected. As such, each sample likely represents multiple defecation bouts per animal per night (Hodges, 1999).

### Experimental approach

All faecal pellet samples were dried for 48 h at 80°C (Möstl *et al.*, 2005). The two samples per individual were randomly assigned to one of two experimental sampling treatment groups. Samples assigned to Group 1 (G1) were first subdivided into five equal portions of intact pellets based on the total dry mass, resulting in five G1 replicates per individual that were separately homogenized using a glass mortar and pestle. For samples assigned to Group 2 (G2), samples were first thoroughly homogenized using a glass mortar and pestle, and subsequently subdivided into five equal portions of powdered faeces based on total dry mass, resulting in five G2 replicates per individual.

### FGM extraction and quantification

From thoroughly homogenized G1 and G2 subsamples, we removed a 0.15 mg subsample and placed it in 15 ml centrifuge tube with 5.0 ml of 80% methanol. Next, we shook samples on a hand vortex for 1 min and centrifuged each sample at 2500 g for 15 min (Palme *et al.*, 2013). Following centrifugation, 0.5 ml of supernatant was transferred to micro-centrifuge tubes and dried for ~2 h at 80°C (Palme *et al.*, 2013). Samples were shipped to the Department of Biomedical Sciences, Unit of Physiology, Pathophysiology and Experimental Endocrinology at the University of Veterinary Medicine in Vienna, Austria for EIA analysis. FGM concentrations were quantified in the re-dissolved extracts using an 11-oxoetiocholanolone-EIA (Palme, 2005; Rehnus *et al.*, 2009; Sheriff *et al.*, 2009a).

### Data analysis

To quantify within-sample variation in FGM concentrations, we compared the coefficient of variation (CV) for G1 and G2 for each individual using a paired *t*-test. In addition, to facilitate future sampling efforts we conducted a power analysis to determine the sample size necessary to detect differences of 10%, 20%, 30%, 40% and 50% in FGM based on the percent differences and degree of variation we observed. We fixed statistical power at 0.80 and *α* at 0.05. All statistical analyses were conducted in R 3.2.4 (R Core Team, 2014).

## Results

**Figure 1:**
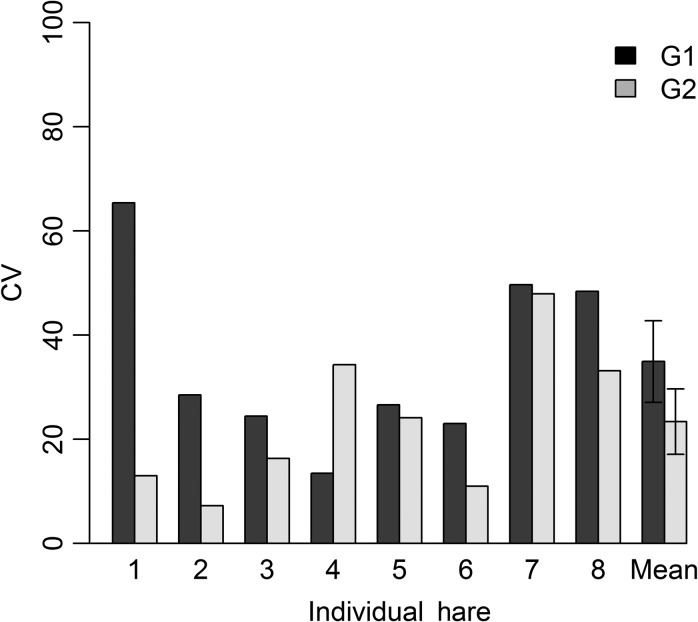
Percent CV in faecal cortisol metabolite concentration based on five repeated measures from each individual hare and overall mean (Mean) of all hares for two sampling techniques. Group 1 (G1) consisted of whole samples of intact pellets that were subsequently subdivided into five equal portions of intact pellets based on the total dry mass; Group 2 (G2), the whole samples were first thoroughly homogenized and subsequently subdivided into five equal portions of powdered faeces based on total dry mass. Standard error indicated with error bars.

**Figure 2:**
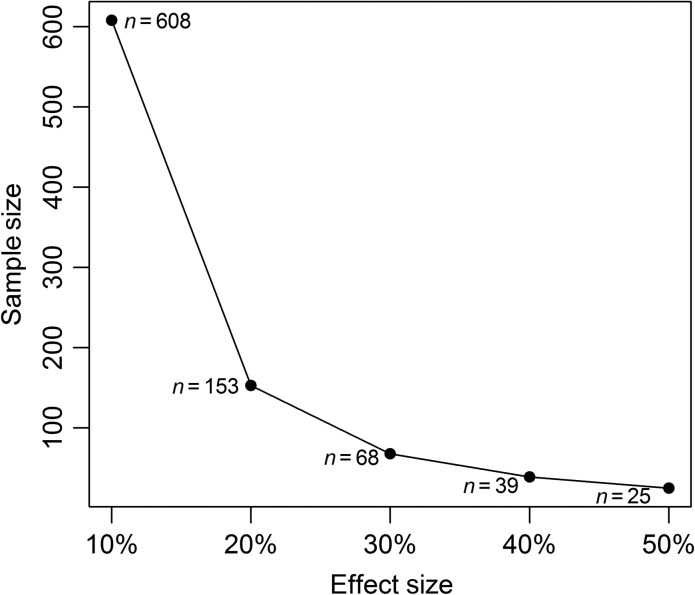
Necessary sample size to detect a difference (10%, 20%, 30%, 40% and 50%) in faecal cortisol metabolite concentrations when whole samples were first thoroughly homogenized and subsequently subdivided into five equal portions of powdered faeces based on total dry mass (G2). Statistical power was fixed at 0.80 and α = 0.05.

Within-sample variation in FGM measurements represented by mean %CV was slightly lower for sample processing technique G2 (%CV 23, range 7.26–47.94) compared to G1 (%CV = 35, range 13.45–65.37), although the mean difference was not significant (two sample *t*-test, *n* = 8, *t* = 1.57, *P* = 0.16; Fig. 1). This EIA had an intra-assay CV of 3.7% and the inter-assay coefficients of variation for high- and low-concentration standards across plates were 8.9% and 14.1%. We based our power analysis on the technique with the slightly lower CV (G2: homogenized complete sample). Based on variance estimates from our experiments, ~600 faecal pellet samples would be necessary to detect a 10% difference in FGM concentrations, whereas an effect of 20% would only require ~150 samples (Fig. 2).

## Discussion

Based on our finding of no significant differences in %CV between our two sampling techniques, FGM appears to be relatively evenly distributed throughout snowshoe hare faecal pellet groups. The %CV we calculated for FGM derived from snowshoe hare pellets was higher than %CV calculated in other studies of distribution of steroid metabolites in faeces (Brown *et al.*, 1994; Wasser *et al.*, 1996; Millspaugh and Washburn, 2003), although such differences among studies are not surprising given species-specific and methodological differences (Millspaugh and Washburn, 2003). Although not statistically different, we found a slightly lower %CV for subsamples taken from homogenized whole pellet groups (G2) compared to subsamples comprised of a few intact faecal pellet (G1).

Because our goal was to estimate the precision of FGM estimates from a single sample and to determine sample-size requirements for detecting differences in FGM concentrations among treatments, it is important to consider factors that may contribute to the levels of within-sample variation observed in our study. First, our sampling regime was designed to reflect a time frame (e.g. 10 h) similar to field studies where pellets are collected from individual snowshoe hares in live traps. Because captive snowshoe hares may defecate several times during this time interval (Hodges, 1999, Lafferty pers. obs), within-sample variability in FGM concentrations may be the result of subsamples that reflect hormonal fluctuations associated with both the circadian rhythm (Rich and Romero, 2001; Carere *et al.*, 2003; Sheriff *et al.*, 2009a) and adrenocortical responses over shorter time periods. Thus, subsampling a few snowshoe hare faecal pellets from the whole pellet group may capture specific points in the diel cycle that contain lower or higher FGM concentrations compared to the average concentration of FGM for the whole faecal pellet group (see Sherrif *et al.*, 2009a). Millspaugh and Washburn (2003) reported similar findings in which analysis of a few selected white-tailed deer (*Odocoileus virginianus*) pellets resulted in greater variability in FGM concentrations compared to subsamples taken from homogenized whole pellet groups. Results from previous studies also suggest that diet as well as the size of the faecal mass produced can influence within-sample variability in FGM concentrations (Wasser *et al.*, 1993; Brown *et al.*, 1994; Sheriff *et al.*, 2009a). Millspaugh and Washburn (2003) posited that low %CV in white-tailed deer faecal pellets compared to other species may be the result of a fairly homogeneous diet and relatively small volume of faeces produced. Although fed a consistent pelleted commercial diet during this study, snowshoe hares produce a large amount of faeces relative to their body weight. Measures of FGM concentration from field-derived samples may result in greater levels of within-sample variation than we found during our controlled laboratory experiment, due to greater exposure to a range of abiotic and biotic environmental conditions and potentially less control over the temporal sampling frame (Wilkening *et al.*, 2016). As such, FGM measures derived from subsamples of faecal pellet groups should be considered sample estimates that represent a range of potential FGM values, not point values without error.

Findings from our study and others indicate that sampling faeces in a systematic manner as well as standardized processing is critical when collecting samples for FGM analysis, particularly for comparative studies (Wilkening *et al.*, 2016). Our work demonstrates that whether subsampling—faeces or collecting and homogenizing whole faecal pellet groups or a whole faecal mass, researchers should determine *a priori* the sampling protocol that will be used throughout a study to avoid introducing a controllable source of variation to the data (e.g. measurement error) that can bias FGM measurements (Jeffcoate, 1981; Millspaugh and Washburn, 2003). And while collecting a subsample of faeces may be preferential in some situations, collecting and homogenizing whole pellets groups or entire faecal masses (e.g. larger volume samples) may be more appropriate when the sampling time frame is less controlled because a subsample derived from a homogenized whole pellet group or whole faecal mass might be more representative of the animal's ‘average’ psycho-physiological state compared to FGM concentrations derived from a few haphazardly collected pieces of faeces.

Further, we encourage *a priori* consideration of how sample sizes and within-sample variation in FGM will influence the detection of treatment effects. For example, if effect sizes are relatively small (e.g. <20%) the sampling requirement for obtaining sufficient power to detect a statistically significant differences in FGM may be logistically and/or cost prohibitive. However, for many situations of ecological or conservation interest, treatment effects may be quite substantial (e.g. ~25% to >100%) (Hik *et al.*, 2001; Sheriff *et al.*, 2009b, 2011b; Creel *et al.*, 2013a) and can therefore be detected with fewer samples. For example, Sheriff *et al.* (2009b) documented an increase of 214% and 837% in FGM concentrations among female snowshoe hare experimentally exposed to predation risk relative to a control group not exposed to predation risk. In studies of lions (*Panthera leo*), Creel *et al.* (2013a) found 25% differences in FGM concentrations based on land use status and 22% decreases per kilometre with increasing distance from human settlement (Creel *et al.*, 2013a). In such cases with relatively large effect sizes, more moderate sample sizes may be sufficient for testing research hypotheses regarding FGM concentrations.

Although non-invasive measures of stress are being used increasingly to glean insight into the health and well-being of captive and free-roaming wildlife, much remains unknown of the factors that influence measures of FGM concentrations. For example, species-specific insights regarding the linkages among steroid hormone metabolites and diet (Wasser *et al.*, 1993; Stetz *et al.*, 2013), reproductive state (Sapolsky, 1987; Weingrill *et al.*, 2004; Dantzer *et al.*, 2010), body condition (Kitaysky *et al.*, 1999; Macbeth *et al.*, 2012) and circadian rhythmicity (Breuner *et al.*, 1999; Carere *et al.*, 2003; Sheriff *et al.*, 2009a) would enhance the utility of FGM as a tool for wildlife ecology, conservation and management. Nevertheless, non-invasive measures of endocrine activity have tremendous potential to aid our understanding of wildlife responses to environmental change, enhance wildlife health monitoring, and contribute to the conservation of free-ranging wildlife populations (Madliger *et al.*, 2016). With further refinement of sampling methodologies, measures of FGM concentrations will be a powerful non-invasive tool for assessing and mitigating the impacts of environmental stressors on wildlife health at both the individual and population level.
